# Supplementary material for: Rewiring MAP kinases in Saccharomyces cerevisiae to regulate novel targets through ubiquitination
Source: eLife. 2016 Aug 15;5:e15200. doi: 10.7554/eLife.15200 (PMC5019841; doi:10.7554/eLife.15200)
Supplement: Supplementary file 1. — DOI: http://dx.doi.org/10.7554/eLife.15200.021 [file elife-15200-supp1.docx]

**Table of all yeast strains:**

| **Strain database number** | **Integrated constructs** | **Used in figure** |
| --- | --- | --- |
| **DK2 + DK8** | **URA - pGPD:FUS3-12xGSlinker-PDZligand**  **TRP – pGPD:SV40-12xGSlinker-YFP-12xGSlinker-Tec1Phosphodegron-mPDZ** | **1,2,3** |
|  | **URA - pGPD:FUS3-12xGSlinker-PDZligand**  **TRP – pGPD:SV40-12xGSlinker-YFP-12xGSlinker-Tec1MutantPhosphodegron-mPDZ** | **1,2** |
|  | **URA - pGPD:FUS3(Kinase Dead)-12xGSlinker-PDZligand**  **TRP – pGPD:SV40-12xGSlinker-YFP-12xGSlinker-Tec1MutantPhosphodegron-mPDZ** | **1** |
|  | **URA - pGPD:FUS3-12xGSlinker-PDZligand**  **TRP – pGPD:SV40-12xGSlinker-YFP-12xGSlinker-Tec1Phosphodegron-yoSH3** | **1** |
|  | **In RG565 (MG132 sensitized) Yeast background**  **URA - pGPD:FUS3-12xGSlinker-PDZligand**  **HIS – pGPD:SV40-12xGSlinker-YFP-12xGSlinker-Tec1Phosphodegron-mPDZ** | **1.2 (supplement)** |
|  | **In RG565 (MG132 sensitized) Yeast background**  **URA - pGPD:FUS3-12xGSlinker-PDZligand**  **HIS – pGPD:SV40-12xGSlinker-YFP-12xGSlinker-Tec1MutantPhosphodegron-mPDZ** | **1.2 (supplement)** |
|  | **URA - pGPD:FUS3-12xGSlinker-mPDZligand**  **TRP – pGPD:SV40-12xGSlinker-YFP-12xGSlinker-Tec1Phosphodegron-PDZligand** | **1.3 (supplement)** |
|  | **TRP – pGPD:SV40-12xGSlinker-YFP-12xGSlinker-Tec1Phosphodegron-mPDZ** | **1.4 (supplement)** |
|  | **FUS3(URA) – FUS3(nativeLocus)- 12xGSlinker-PDZligand**  **TRP – pGPD:SV40-12xGSlinker-YFP-12xGSlinker-Tec1MutantPhosphodegron-mPDZ** | **1.4 (supplement)** |
|  | **FUS3(URA) – FUS3(nativeLocus)- 12xGSlinker-PDZligand**  **TRP – pGPD:SV40-12xGSlinker-YFP-12xGSlinker-Tec1Phosphodegron-mPDZ** | **1.4 (supplement)** |
| **DK1 + DK6** | **URA - pGPD:FUS3-12xGSlinker-PYL**  **TRP – pGPD:SV40-12xGSlinker-YFP-12xGSlinker-Tec1Phosphodegron-ABI** | **2C** |
| **DK16 + DK6** | **URA - pGPD:FUS3-12xGSlinker-ABI**  **TRP – pGPD:SV40-12xGSlinker-YFP-12xGSlinker-Tec1MutantPhosphodegron-PYL** | **2C** |
| **DK3 + DK10** | **URA - pGPD:FUS3-12xGSlinker-SYNZIP6**  **TRP – pGPD:SV40-12xGSlinker-YFP-12xGSlinker-Tec1Phosphodegron-SYNZIP5** | **2B** |
| **DK18 + DK10** | **URA - pGPD:FUS3-12xGSlinker-SYNZIP6**  **TRP – pGPD:SV40-12xGSlinker-YFP-12xGSlinker-Tec1MutantPhosphodegron-SYNZIP5** | **2B** |
| **DK5 + DK15** | **URA - pGPD:SH3ligand-12xGSlinker-FUS3**  **TRP – pGPD:SV40-12xGSlinker-YFP-12xGSlinker-Tec1Phosphodegron-SH3** | **2B** |
| **DK20 + DK15** | **URA - pGPD:SH3ligand-12xGSlinker-FUS3**  **TRP – pGPD:SV40-12xGSlinker-YFP-12xGSlinker-Tec1MutantPhosphodegron-SH3** | **2B** |
| **9820** | **URA - pGPD:FUS3-12xGSlinker-PYL**  **TRP – pGPD:SV40-12xGSlinker-YFP-12xGSlinker-Tec1Phosphodegron-ABI**  **HIS - pGPD:FUS3-12xGSlinker-PDZligand**  **Leu – pGPD:SV40-12xGSlinker-mCherry-12xGSlinker-Tec1Phosphodegron-mPDZ** | **3** |
|  | **URA - pGPD:FUS3-12xGSlinker-PYL**  **TRP – pGPD:SV40-12xGSlinker-YFP-12xGSlinker-Tec1Phosphodegron-ABI**  **HIS – pGPD:FUS3-12xGSlinker-PDZligand** | **3.1 (supplement)** |
|  | **URA - pGPD:FUS3-12xGSlinker-PDZligand**  **TRP – pGPD:SV40-12xGSlinker-mCherry-12xGSlinker-Tec1Phosphodegron-mPDZ**  **LEU – pGPD:SV40-12xGSlinker-YFP-12xGSlinker-Tec1Phosphodegron-mPDZ** | **3.2 (supplement)** |
|  | **URA - pGPD:FUS3-12xGSlinker-PDZligand**  **TRP – pGPD:SV40-12xGSlinker-YFP-12xGSlinker-(2x)Tec1Phosphodegron-mPDZ** | **4B** |
| **4913** | **URA - pGPD:FUS3-12xGSlinker-PDZligand**  **TRP – pGPD:SV40-12xGSlinker-YFP-12xGSlinker-(3x)Tec1Phosphodegron-mPDZ** | **4B** |
| **4914** | **URA - pGPD:FUS3-12xGSlinker-PDZligand**  **TRP – pGPD:SV40-12xGSlinker-YFP-12xGSlinker-(4x)Tec1Phosphodegron-mPDZ** | **4B** |
| **3145** | **URA - pGPD:FUS3-12xGSlinker-PDZligand**  **TRP – pGPD:SV40-12xGSlinker-YFP-12xGSlinker-(5x)Tec1Phosphodegron-mPDZ** | **4B** |
|  | **URA - pGPD:FUS3-12xGSlinker-PDZligand**  **TRP – pGPD:SV40-12xGSlinker-YFP-12xGSlinker-Variant1Phosphodegron-mPDZ** | **4C** |
|  | **URA - pGPD:FUS3-12xGSlinker-PDZligand**  **TRP – pGPD:SV40-12xGSlinker-YFP-12xGSlinker-Variant2Phosphodegron-mPDZ** | **4C** |
| **5967** | **URA - pGAL(Z4):ERK2-MEK1-PDZligand**  **TRP - pGPD:SV40-12xGSlinker-YFP-MKP1Phosphodegron –mPDZ**  **HO(Kan) - pACT1:ZEV4** | **5** |
| **5966** | **URA - pGAL(Z4):ERK2-MEK1-PDZligand**  **TRP - pGPD:SV40-12xGSlinker-YFP-MKP1MutantPhosphodegron –mPDZ**  **HO(Kan) - pACT1:ZEV4** | **5** |
| **6386** | **URA - pGAL(Z4):ERK2-MEK1-STOP-PDZligand**  **TRP - pGPD:SV40-12xGSlinker-YFP-MKP1Phosphodegron –mPDZ**  **HO(Kan) - pACT1:ZEV4** | **5** |
| **6387** | **URA - pGAL(Z4):ERK2-MEK1-STOP-PDZligand**  **TRP - pGPD:SV40-12xGSlinker-YFP-MKP1MutantPhosphodegron –mPDZ**  **HO(Kan) - pACT1:ZEV4** | **5** |
| **3303** | **HIS - pGPD:FUS3-12xGSlinker-PDZligand**  **STE12(URA) – pIL-STE12-mCherry-PDZ-STOP**  **FUS1 – pFUS1:YFP** | **6** |
| **3304** | **HIS - pGPD:FUS3-12xGSlinker-PDZligand**  **STE7(URA) – pIL-STE7-mCherry-PDZ-STOP**  **FUS1 – pFUS1:YFP** | **6** |
| **3305** | **HIS - pGPD:FUS3-12xGSlinker-PDZligand**  **STE5(URA) – pIL-STE5-mCherry-PDZ-STOP**  **FUS1 – pFUS1:YFP** | **6** |
| **4980** | **HIS - pGPD:FUS3-12xGSlinker-SYNZIP6**  **STE12(URA) – pIL-STE12-mCherry-PDZ-STOP**  **FUS1 – pFUS1:YFP** | **6** |
| **4978** | **HIS - pGPD:FUS3-12xGSlinker-SYNZIP6**  **STE7(URA) – pIL-STE7-mCherry-PDZ-STOP**  **FUS1 – pFUS1:YFP** | **6** |
| **4977** | **HIS - pGPD:FUS3-12xGSlinker-SYNZIP6**  **STE5(URA) – pIL-STE5-mCherry-PDZ-STOP**  **FUS1 – pFUS1:YFP** | **6** |
| **8401** | **URA - pGPD:FUS3-12xGSlinker-PDZligand**  **HIS – pFUS1(till -435):SV40-12xGSlinker-YFP-12xGSlinker-Tec1Phosphodegron-mPDZ** | **7** |
| **8402** | **URA - pGPD:FUS3-12xGSlinker-PDZligand**  **HIS – pFUS1(till -435):SV40-12xGSlinker-YFP-12xGSlinker-Tec1MutantPhosphodegron-mPDZ** | **7** |
